# Comparison of Thiol-Disulfide Hemostasis With Inflammatory Markers in the Inflammatory Process of Acute Appendicitis

**DOI:** 10.7759/cureus.28891

**Published:** 2022-09-07

**Authors:** Abdulkadir Ünsal, Alper Yavuz, Hakan Buluş, Altan Aydın, Murat Alışık, Özcan Erel

**Affiliations:** 1 General Surgery, University of Health Sciences, Atatürk Sanatoryum Training and Research Hospital, Ankara, TUR; 2 General Surgery, University of Health Sciences, Trabzon Kanuni Training and Research Hospital, Trabzon, TUR; 3 Department of Clinical Chemistry, Yıldırım Beyazıt University, Ankara, TUR; 4 Department of Clinical Chemistry, Yıldırım Beyazit University, Ankara, TUR

**Keywords:** acute, systemic inflammatory markers, thiol-disulfide, thiol, appendicitis

## Abstract

Background

Acute appendicitis is one of the events most frequently encountered by general surgeons. Despite the high incidence, serious problems are experienced in the diagnosis and clinical follow-up. In the pathogenesis of the disease, oxidative stress and impaired antioxidant defense mechanisms created in the body by this stress play an important role. As dynamic thiol-disulfide hemostasis is closely related to oxidative stress and is known to have a crucial role in the pathogenesis of oxidative stress, this study aimed to compare its value with other inflammatory markers in the diagnosis and follow-up of acute appendicitis.

Methodology

This study included cases admitted for surgery with a diagnosis of acute abdomen at Keçiören Research and Training Hospital General Surgery Clinic between April 2015 and July 2015 who were intraoperatively diagnosed with acute appendicitis and underwent routine appendectomy. In the preoperative period and after clinical healing before discharge, blood samples were obtained to examine white blood cell (WBC), mean platelet volume (MPV), total bilirubin, C-reactive protein (CRP), and thiol-disulfide balance, and the results were recorded.

Results

A total of 68 cases were operated on for acute appendicitis, and 59 were evaluated comprising 23 (39%) females and 36 (61%) males with a mean age of 35.6 years (range = 19-65 years). The mean duration of hospital stay was two days (range = 1-8 days). The results of the tests performed preoperatively and before discharge and their p-values were as follows: native thiol (-SH) 393.5 ± 9.4 µmol/L and 369.3 ± 9.5 µmol/L (p = 0.04), total thiol 434 ± 9.7 µmol/L and 396.7 ± 10.2 µmol/L (p = 0.03), disulfide (-S-S) 16.8 ± 0.7 µmol/L and 15.7 ± 0.9 µmol/L (p = 0.3), WBC 13.2 ± 0.5 × 10³/mL and 9.2 ± 0.4 × 10³/mL (p = 0.0), CRP 8.17 ± 1.24 mg/L and 7.84 ± 0.82 mg/L (p = 0.17), MPV 7.4 ± 0.37 fL and 7.97 ± 0.19 fL (p = 1.0), and total bilirubin 0.86 ± 0.08 mg/dL and 0.69 ± 0.06 mg/dL (p = 0.08).

Conclusions

In the clinical follow-up of acute appendicitis patients, the decrease in WBC, total thiol, and native thiol values can be helpful to clinicians as markers of clinical healing. However, CRP may not be a useful marker of clinical healing in acute appendicitis patients who are discharged early.

## Introduction

Acute appendicitis is one of the most common reasons for acute abdomen diseases, and appendectomy is one of the most frequently performed operations by general surgeons [1]. Despite its frequent occurrence, serious difficulties are experienced in diagnosis and follow-up [2]. In the current treatment of acute appendicitis, medical treatment, in particular, has been much debated, demonstrating its importance in diagnosis and follow-up [3,4]. Although the diagnosis of the disease demands clinical evaluation, it is often not sufficient. Although significant technological developments in radiological imaging have facilitated the surgeon’s work, use may be limited because of radiation exposure, cost, and availability. Hence, laboratory tests are always helpful to clinicians as they are cheap, practical, and can be accessed at any time of the day. However, when scoring systems are examined, it can be seen that apart from white blood cell (WBC) count, other laboratory values are not of great help to clinicians.

Thiol components are known to have anti-oxidant effects in the body and are formed of disulfide bonds (RSSR) entering an oxidation reaction against oxidants. Oxidative stress among thiol groups may lead to the formation of a mixed disulfide structure. These disulfides can again be reduced to thiol groups, and thus the dynamic thiol-disulfide hemostasis can be maintained. Oxidative stress is known to play a crucial role in the pathogenesis of dynamic thiol-disulfide hemostasis which has a close relationship with oxidative stress; therefore, it has been considered that it can be critical in the diagnosis and follow-up of acute appendicitis [5].

This study aimed to compare the dynamic thiol-disulfide values of patients admitted to the hospital with acute appendicitis with other laboratory parameters (WBC, C-reactive protein (CRP), bilirubin, mean platelet volume (MPV)) which can be helpful in monitoring the inflammatory process, both on admission and before discharge from the hospital.

## Materials and methods

This prospective clinical study included cases admitted for surgery with a diagnosis of acute abdomen at Keçiören Research and Training Hospital General Surgery Clinic between April 2015 and July 2015 who were intraoperatively diagnosed with acute appendicitis and underwent routine appendectomy. The study was approved by the Ethics Committee on Clinical Research of Ankara Atatürk Sanatorium Teaching and Research Hospital (2015-KAEK-B.10.4.İSM.4.06.68.49). The diagnosis of acute appendicitis was made with clinical symptoms and physical examination as well as laboratory tests and imaging techniques such as ultrasonography and computed tomography. The intraoperative appendix perforation status was recorded. Patients were excluded if they were over 65 years of age, if they had a chronic disease (diabetes, chronic obstructive pulmonary disease, hypertension, cardiac disease), if a procedure apart from routine appendectomy was performed, or if appendicitis was not observed in the pathological examination. During the preoperative period and to monitor the inflammatory process after clinical healing before discharge, blood samples were obtained to examine hemogram (WBC, MPV), biochemistry (total bilirubin), serology (CRP), and thiol-disulfide balance, and the results were recorded. A record was made of the duration of hospital stay and events which prolonged the hospitalization during the clinical follow-up.

The basis of thiol-disulfide homeostasis is that dynamic disulfide bonds (-S-S-) are broken down to functional thiol groups (-SH) by sodium borohydride. In the assay, the sodium borohydride remnants were removed with formaldehyde. The total thiol content of the sample was assayed using the modified Ellman reagent. The native thiol content was calculated from the total thiol content and half of the acquired difference provided the disulfide bond amount. The native thiol-disulfide ratio (-S-S-/-SH) was calculated with native thiol (-SH) and the disulfide (-S-S) amount.

Statistical evaluation was done using SPSS version 18 software (SPSS Inc. Chicago, IL, USA). Conformity to normal distribution of the variables on admittance to the hospital and before discharge was examined using visual and analytical methods. When values showed normal distribution (total thiol, native thiol, disulfide, WBC), the comparison was done using the Student’s t-test. Values that did not show normal distribution (CRP, MPV, total bilirubin) were compared using the Wilcoxon test. P-values of <0.05 were accepted as statistically significant.

## Results

A total of 68 cases were admitted for acute appendicitis operation. A total of nine cases were excluded; in four cases, procedures other than routine appendectomy were performed because of complicated appendicitis, and in five cases, pathologies other than appendicitis were observed. Of the remaining 59 cases for evaluation, 23 (39%) were female and 36 (61%) were male, with a mean age of 35.6 years (range = 19-65 years) (Table 1). In the appendicitis group, 11.9% (n = 7) of the patients had perforated and 88.1% (n = 52) had non-perforated appendicitis. Appendectomy was performed with laparoscopic technique in 33.89% (n = 20) and open technique in 66.1% (n = 39) of patients.

**Table 1:** Clinical features of cases.

Parameters	
Age, mean (range)	35.6 (19–65)
Sex, n (%)	
Female	23 (39)
Male	36 (61)
Excluded cases, n (%)	
Surgical operations other than routine appendectomy	4 (5.9)
Pathological examination revealed no appendicitis	4 (7.4)
Total	9 (13.2)
Type of appendicitis, n (%)	
Non-perforated	52 (88.1)
Perforated	7 (11.9)
Hospital stay, days (range)	2 (1–8)

The mean duration of hospital stay was two days (range = 1-8 days). The results of the tests performed preoperatively and before discharge and their p-values were as follows: native thiol (SH) 393.5 ± 9.4 µmol/L and 369.3 ± 9.5 µmol/L (p = 0.04), total thiol 434 ± 9.7 µmol/L and 396.7 ± 10.2 µmol/L (p = 0.03), disulfide (-S-S) 16.8 ± 0.7 µmol/L and 15.7 ± 0.9 µmol/L (p = 0.3), WBC 13.2 ± 0.5 × 10³/mL and 9.2 ± 0.4 × 10³/mL (p = 0.0), CRP 8.17 ± 1.24 mg/L and 7.84 ± 0.82 mg/L (p = 0.17), MPV 7.4 ± 0.37 fL and 7.97 ± 0.19 fL (p = 1.0), and total bilirubin 0.86 ± 0.08 mg/dL and 0.69 ± 0.06 mg/dL (p = 0.08) (Table 2).

**Table 2:** Laboratory test results measured on admission to the hospital and before discharge. WBC: white blood cell; CRP: C-reactive protein; MPV: mean platelet volume

	Native thiol (µmol/L)	Total thiol (µmol/L)	Disulfide (µmol/L)	WBC (×10³/mL)	CRP (mg/L)	MPV (fL)	Total bilirubin (mg/dL)
On admission	393.5 ± 9.4	434 ± 9.7	16.8 ± 0.7	13.2 ± 0.5	8.17 ± 1.24	7.4 ± 0.37	0.86 ± 0.08
Before discharge	369.3 ± 9.5	396.7 ± 10.2	15.7 ± 0.9	9.2 ± 0.4	7.84 ± 0.82	7.97 ± 0.19	0.69 ± 0.06
P-value	0.04	0.03	0.3	0.0	0.17	1.0	0.08

## Discussion

Oxidative stress occurs because of the imbalance between oxidants and antioxidants and plays a role in the pathogenesis of several diseases. Although few in number, studies have shown oxidative stress to play a role in the pathogenesis of acute appendicitis [6-8]. Previous studies have shown that the active thiol-disulfide balance, which is closely related to oxidative stress, plays a role in the pathogenesis of several diseases. In this study, the status of the inflammatory process in acute appendicitis was examined with the dynamic thiol-disulfide balance and other laboratory tests (WBC, CRP, MPV, total bilirubin) at the time of admission to the hospital and before discharge.

In a clinical study by Köksal et al., total oxidant status (TOS) and total antioxidant status (TAS) were evaluated to define oxidative stress in patients with acute appendicitis. Both the TOS and TAS values were observed to be higher in acute appendicitis patients compared to the control group [6]. Similarly, Koltuksuz et al. determined that superoxide dismutase (oxidant) and malondialdehyde (antioxidant) values were high in patients with acute appendicitis compared to the control group. This was explained as possibly due to the progression of the inflammation and the response of the body to this oxidative stress [7]. In this study, there was a significant increase in the native thiol and total thiol values measured on admission to the hospital compared with the values noted before discharge.

Although not statistically significant, there was also an increase in the disulfide values. That the difference in the disulfide values was not significant can be attributed to not having included advanced-stage complicated appendicitis in this study.

CRP is an acute-phase reactant used in the diagnosis and follow-up of several diseases, primarily inflammatory diseases. This plasma protein, which is produced in the liver, increases rapidly as a response to wounds, infections, and other inflammatory stimuli in the body [9,10]. Elevated CRP is not specific to a disease but diseases that can stimulate the inflammatory process should come to mind immediately when there is an increase in CRP. As the severity of the inflammatory process increases, the CRP level also increases, and the diagnostic value in these advanced inflammatory diseases is higher. However, even when the inflammatory process recedes, this protein level, which has a long half-life of up to 19 hours, may remain high for a long period. Previous studies have reported that even after problem-free operations, CRP values may be high for four to five days [9].

In the literature, there is no consensus on the use of CRP in the diagnosis of acute appendicitis, but it has been emphasized that CRP can be helpful in the diagnosis of advanced and complicated appendicitis [11,12]. In this study, the mean CRP values of patients on admission to the hospital (8.17 ± 1.24 mg/L) were above the normal limit (5 mg/L). With the clinical recovery of the patients, the CRP values measured before hospital discharge (7.84 ± 0.82 mg/L) were lower than those on admission but the decrease was not statistically significant. Furthermore, the mean CRP values before discharge from the hospital were also above the normal upper limit.

It has been reported that in the inflammatory process, serum bilirubin levels are increased without any liver or cholestatic disease. The mechanism of the increase has been attributed to cholestasis which forms due to bacterial endotoxins or increased bilirubin expression through the effect on the hepatobiliary system of oxidative stress, which affects many systems in the body. Although several studies in the literature have shown a correlation between elevated bilirubin in acute appendicitis and the severity of the disease, it has been emphasized that bilirubin alone may not be sufficient for the diagnosis of cases in the early stages [13,14]. In this study, the mean total bilirubin values of the patients on admission to the hospital (0.86 ± 0.08 mg/dL) were higher than the mean values measured before hospital discharge (0.69 ± 0.06 mg/dL). However, this difference was not statistically significant (p = 0.08). The total bilirubin levels of the patients with acute appendicitis in this study were within the range of the normal laboratory values (0.3-1.3 mg/dL). Therefore, the total bilirubin values were of no benefit in the monitoring of the inflammatory process.

MPV showing thrombocyte dimensions is routinely used in complete blood count (CBC) analyses but is a test that is generally overlooked by clinicians. It has been shown in the literature that the MPV value is affected in the inflammatory process, just as in many diseases. Bozkurt et al. investigated the diagnostic value of MPV in acute appendicitis. Although no significant difference was observed in the MPV values between the control and appendicitis groups, a lower MPV value was observed in complicated appendicitis patients compared to the control or non-complicated groups [15]. In this study, the mean MPV value of patients on admission to the hospital was 7.4 ± 0.37 fL. This value was below the normal value (7.8-11 fL) and consistent with the literature; the MPV value was low at the time of diagnosis. There was an increase in the MPV values before hospital discharge, and with this increase, the mean MPV value (7.4 ± 0.37 fL) was seen to be within normal limits. However, the increase observed in the MPV value together with clinical recovery was not statistically significant.

The inflammatory biomarker most commonly used by clinicians in the diagnosis of acute appendicitis is WBC. However, as reported by Yokoyama et al., some clinicians believe that WBC is not an indicative factor for surgery [16]. Given that elevated WBC in clinical application leads to several events and diseases and WBC may not be elevated in acute appendicitis, the clinician’s work becomes more difficult. In this study, the WBC values were observed to be high in acute appendicitis cases (13.2 ± 0.5 × 10³/mL), and together with clinical recovery, a decrease (9.2 ± 0.4 × 10³/mL) was observed in the values. This decrease seen together with clinical recovery was statistically significant (p = 0.0).

In previous studies concerning acute appendicitis and laboratory tests, the research has generally focused on diagnosing the disease or determining its severity. However, when medical treatment has been debated in the current treatment of the disease, the determination of the clinical course demands as much importance as the determination of the severity of the disease. Okuş et al. evaluated the CRP and WBC values in acute appendicitis patients where medical treatment was not successful. A substantial increase was determined in the CRP values of patients with unsuccessful treatment [4]. However, overreliance on a laboratory test with a long half-life can lead to serious problems in critical patients. In the clinical evaluation of the CRP value, as reported by Okuş et al., it may be appropriate to take into consideration the use of a high cut-off value of CRP such as 80.8 mg/dL in the evaluation of unsuccessful treatment; however, in cases of clinical recovery, the slow decrease in the CRP value may leave physicians in doubt. In such cases, the decrease in thiol and WBC values as an indicator of clinical recovery can be helpful to the physician on the subject of clinical recovery.

Our study was designed as a prospective study. Therefore, more reliable data have been obtained compared to various retrospective studies. There are several limitations in this study that should be taken into consideration. First is the inclusion of a relatively small number of patients who were admitted to a single center. Therefore, studies with a large number of participants can provide more valuable results. Other diagnostic aids such as procalcitonin and imaging techniques such as ultrasonography and/or computed tomography and Alvarado score were not correlated with thiol/disulfide homeostasis parameters.

## Conclusions

Although acute appendicitis is frequently encountered by general surgeons, serious problems are experienced in diagnosis and follow-up. This study demonstrated that dynamic thiol/disulfide homeostasis shifted toward disulfide formation as a result of thiol oxidation in patients with acute appendicitis. A thorough clinical examination which is correlated with laboratory tests plays a significant role in the clinical follow-up. In the determination of the status of clinical recovery, reductions in total thiol, native thiol, and WBC values are important. However, the benefit is not provided at a sufficient level from CRP, which is a test with a long half-life, as a significant reduction was not achieved in this process. Prospective and randomized controlled trials are necessary to confirm the pathophysiologic role of thiol/disulfide homeostasis in acute appendicitis. Further studies are required to optimize the use of this novel oxidative stress marker in conjunction with other established approaches.
